# Self-Healing Thermoplastic Polyurethane Linked via Host-Guest Interactions

**DOI:** 10.3390/polym12061393

**Published:** 2020-06-22

**Authors:** Changming Jin, Garry Sinawang, Motofumi Osaki, Yongtai Zheng, Hiroyasu Yamaguchi, Akira Harada, Yoshinori Takashima

**Affiliations:** 1 Department of Macromolecular Science, Graduate School of Science, Osaka University, 1-1 Machikaneyama-cho, Toyonaka, Osaka 560-0043, Japan; jinc18@chem.sci.osaka-u.ac.jp (C.J.); garrys16@chem.sci.osaka-u.ac.jp (G.S.); osakim16@chem.sci.osaka-u.ac.jp (M.O.); hiroyasu@chem.sci.osaka-u.ac.jp (H.Y.); 2 Project Research Center for Fundamental Sciences, Graduate School of Science, Osaka University, 1-1 Machikaneyama-cho, Toyonaka, Osaka 560-0043, Japan; zhengy16@chem.sci.osaka-u.ac.jp; 3 The Institute of Scientific and Industrial Research, Osaka University, 8-1 Mihogaoka, Ibaraki, Osaka 567-0047, Japan; 4 Institute for Advanced Co-Creation Studies, Osaka University, 1-1 Yamadaoka, Suita, Osaka 565-0871, Japan

**Keywords:** thermoplastic polyurethane, elastomer, tough materials, self-healing, cyclodextrin, host-guest interactions

## Abstract

High toughness with self-healing ability has become the ultimate goal in materials research. Herein, thermoplastic polyurethane (TPU) was linked via host-guest (HG) interactions to increase its mechanical properties and self-healing ability. TPU linked via HG interactions was prepared by the step-growth bulk polymerization of hexamethylene diisocyanate (HDI), tetraethylene glycol (TEG), and HG interactions between permethylated amino βCD (PMeAmβCD) and adamantane amine (AdAm). TPU linked with 10 mol% of HG interactions (HG(10)) showed the highest rupture stress and fracture energy (*G*_F_) of 11 MPa and 25 MJ·m^−3^, which are almost 40-fold and 1500-fold, respectively, higher than those of non-functionalized TEG-based TPU (PU). Additionally, damaged HG(10) shows 87% recovery after heated for 7 min at 80 °C, and completely cut HG(10) shows 80% recovery after 60 min of reattachment at same temperature. The HG interactions in TPU are an important factor in stress dispersion, increasing both its mechanical and self-healing properties. The TPU linked via HG interactions has great promise for use in industrial materials in the near future.

## 1. Introduction

Self-healing polymers are well known for their ability to autonomously repair physical damage [1,2,3,4,5,6,7]. The repairability of self-healing polymers is important in environmental applications, because it increases their lifetime. Self-healing polymers are popular in a variety of fields such as coatings [8], biomedical applications [9,10], and electronic devices [11,12,13,14]. Several self-healing polymers, such as tough polymeric gels [15,16], multiple network gels [17,18], nanocomposite gels [19], four-armed macromolecules [20,21], polyrotaxane [22,23,24,25] or slide-ring gels [26], and doubly cross-linked gels [27], have been reported. These gels are mostly realized using dynamic covalent bonds like Diels-Alder (DA) adducts [28,29], multiple noncovalent bonds, such as hydrogen bonds (H bonds) [30,31], *π-π* stacking interactions [32,33], metal-ligand interactions [34,35,36,37], and host-guest (HG) interactions [38,39,40,41,42,43]. However, self-healing polymers in gels are not considered applicable materials, due to their low mechanical properties. Increasing the self-healing capability and toughness simultaneously in self-healing materials is generally not an easy task, because those properties are contradictory to each other [43,44]. Therefore, another approach, such as using elastomers [45,46,47] as self-healing polymers, has been considered for preparing tough self-healing polymers.

HG interactions, as a kind of noncovalent bond, using cyclodextrins (CDs) as the host units have been popular to use to construct self-healing polymers [48]. Recently, we also reported a self-healing elastomer prepared by bulk polymerization based on HG, using CDs as the host units and suitable guest units [49,50]. Unfortunately, their rupture stress just reached the kPa scale meaning that they cannot be classified as tough materials. Thermoplastic polyurethane (TPU) [51,52,53] as a thermoprocessable elastomer has recently attracted attention in the preparation of tough materials, due to its chemical structure, which can be finely tuned to generate the desired transparency and mechanical properties. TPU usually has a multiphase microstructure, in which the hard domains act as reinforcing filler, and they are connected by thermally reversible crosslinks and embedded in the soft matrix. TPU usually applied as coating, sealant, automotive industry, and footwear. Self-healing properties are interesting to be added in TPU, as they will prolong the lifetime of the materials. For instance, as applied for coating self-healing is very useful that the coating can repair itself when scratched or damaged. Therefore, we prepared mechanically tough and self-healable HG-based elastomers with urethane linkages (HG(*x*)). In addition, HG, as dynamic reversible bonds that result in excellent self-healing efficiency, will support the self-healing ability of TPU.

**Figure 1:**
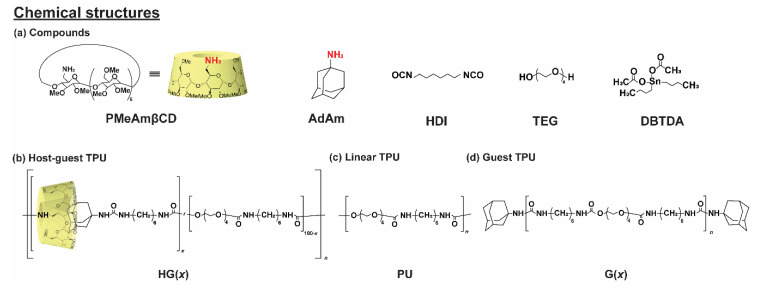
(**a**) Chemical structures of the compounds: permethylated amino βCD (PMeAmβCD), adamantane amine (AdAm), hexamethylene diisocyanate (HDI), tetraethylene glycol (TEG), and dibutyltin diacetate (DBTBA). Chemical structures of thermoplastic polyurethane (TPU) derivatives: (**b**) host-guest (HG) TPU (HG(*x*)), (**c**) linear TPU (PU), and (**d**) guest TPU (G(*x*)). The proportions of the host and guest units in TPU are indicated by “*x*” (HG(*x*) and G(*x*)), which represent the mol% of the PMeAmβCD and AdAm units.

HG(*x*) was prepared by bulk polymerization from hexamethylene diisocyanate (HDI), tetraethylene glycol (TEG), and HG interactions between permethylated amino βCD (PMeAmβCD, Scheme S1, Figures S1–S3) and adamantane amine (AdAm). Figure 1 shows the chemical structures of all the components, HG(*x*), and the reference samples involved: linear TPU (PU) and TPU with only guest monomers (G(*x*)). HG(*x*) exhibited mechanical properties better than those of previously reported host-guest elastomers. While, usually, TPU is constructed from long chain polymer in soft segments and bulky isocyanate, herein, we prepared simple host-guest TPU with short chain soft segments with linear isocyanate. The presence of HG content increased the transparency of TPU, and the increase of the HG content also caused HG(*x*) to show better mechanical properties, more transparency, and a faster self-healing speed.

## 2. Materials and Methods

### 2.1. Materials

Hexamethylene diisocyanate (HDI) and dibutyltin diacetate (DBTDA) were purchased from Tokyo Chemical Industries Co. Ltd., Tokyo, Japan. Tetraethylene glycol (TEG) was obtained from Sigma-Aldrich Co., St. Louis, Missouri, US. 1-Adamantanamine (AdAm) was purchased from Fujifilm Wako Pure Chemical Industries Ltd, Osaka, Japan. Mono-6-*O*-(*p*-toluenesulfonyl)-β-cyclodextrin was obtained from Konan Chemical Industry, Osaka, Japan. All other reagents: triphenylphosphine (PH_3_P), ammonia water (NH_3_·H_2_O), and solvents [tetrahydrofuran (THF), methanol (MeOH), and dichloromethane (CH_2_Cl_2_)] were purchased from Nacalai Tesque Inc, Kyoto, Japan and used without further purification.

### 2.2. Characterizations

The ^1^H, ^13^C and 2D nuclear magnetic resonance (NMR) spectra were recorded with JEOL ECA-400 NMR spectrometer (JEOL Ltd., Tokyo, Japan) at 25 °C. The chemical shifts are referenced to the signal of the solvent such as DMSO-*d*_6_ (δ = 2.49 ppm for ^1^H and δ = 39.51 ppm for ^13^C) and D_2_O (δ = 4.79 ppm for ^1^H). Positive-ion matrix–assisted laser desorption/ionization time-of-flight mass (MALDI-TOF MS) spectrometry was performed by a Bruker autoflex speed mass spectrometer (Bruker, Billerica, Massachusetts, US) using 2,5–dihydroxy–benzoic acid as a matrix. The attenuated total reflectance Fourier-transform infrared spectroscopy (ATR-FTIR) were recorded using JASCO FT/IR-6100 spectrometer (JASCO, Tokyo, Japan) in the wavenumber range from 4000 to 400 cm^−1^ in ATR method. Gel permeation chromatography (GPC) measurements were performed in DMSO (0.40 mL·min^−1^, 25 °C), using a TOSOH HLC-8320GPC EcoSEC^®^ (TOSOH, Tokyo, Japan) equipped with a TOSOH TSK gel α-M column. The UV-Vis absorption spectra were recorded with a JASCO V-650 (JASCO, Tokyo, Japan) in air at room temperature, with an absorption wavelength at 300–800 nm. The 3D micrograph images were taken using a laser scanning confocal microscope VK-X250 (Keyence Co., Osaka, Japan). The mechanical properties (fracture energy, Young’s modulus, and self-healing) were measured using a universal testing machine Autograph AG-X plus (Shimadzu Co., Kyoto, Japan), equipped with a 5 kN load cell with a specific deformation rate of 10 mm·min^−1^ (the sample was prepared in the size of 6 mm × 20 mm × 1 mm). Differential scanning calorimeter (DSC) measurement for glass transition temperatures (*T*_g_) and crystallization temperatures (*T*_c_) of the samples were determined by differential scanning calorimeter DSC7020 System (Hitachi High-Technologies Corporation, Tokyo, Japan) with a heating rate 10 °C·min^−1^.

### 2.3. Preparation of the Host-Guest Thermoplastic Polyurethanes (HG(x))

Predetermined amounts of HG and TEG were vacuum dried at 60 °C for 5 h to remove residual moisture. After cooling down to 4 °C in a refrigerator, HDI and DBTDA were added and the solution was vigorously homogenized. The mixture was rapidly charged into a Teflon mold and allowed to cure at room temperature for 18 h and 70 °C 6 h.

## 3. Results and Discussion

### 3.1. Preparation of Thermoplastic Polyurethane Linked via Host-Guest Interactions

**Figure 2:**
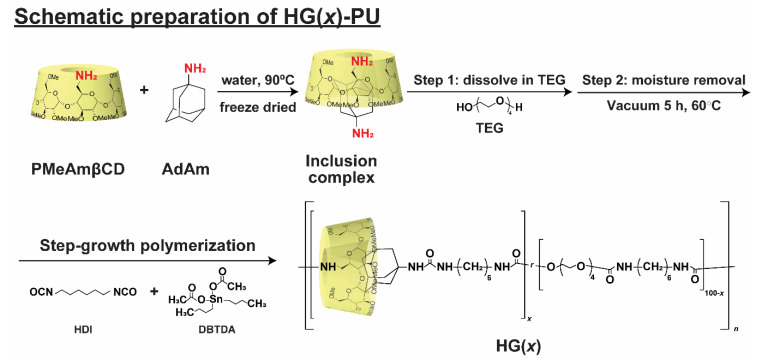
Schematic of the preparation of HG(*x*). The “*x*” in HG(*x*) is the mol% of the PMeAmβCD and AdAm units.

Figure 2 shows the preparation of HG(*x*) using step-growth bulk polymerization. Prior to polymerization, PMeAmβCD and AdAm were mixed in water and lyophilized to obtain an HG inclusion complex powder (Scheme S2, Figures S4–S6). Then, the powder was mixed with TEG at 60 °C under vacuum for 5 h to remove the moisture in the mixture. The mixture was then cooled to 4 °C. Step-growth bulk polymerization was achieved by adding HDI with dibutyltin diacetate (DBTDA) as the catalyst to the mixture and vigorously stirring. Step growth polymerization done in one-stage method, rather than the usual solvent-utilized two-stage method [54], to prevent dissociation of HG caused by solvent during preparation process. To obtain HG(*x*), the mixture was rapidly transferred to a Teflon mold and allowed to cure for 18 h at room temperature (RT) followed by 6 h at 70 °C (Scheme S3, Table S1). To demonstrate the importance of host-guest interactions, PU (Scheme S4, Table S2) and G(*x*) (Scheme S5, Table S3) were also prepared under similar conditions. The proportion of host and guest units in TPU is indicated by “*x*” (HG(*x*) and G(*x*)), which represent the mol% of the PMeAmβCD and AdAm units.

All the prepared TPUs were characterized using ^1^H NMR spectroscopy (Figures S7–S9) and attenuated total Reflectance-Fourier transform infrared (ATR-FTIR) spectroscopy (Figures S10–S12). ^1^H NMR spectroscopy was used to demonstrate the presence of host-guest interactions with urethane and urea linkages in HG(*x*). ATR-FTIR spectroscopy was used to confirm the consumption of the isocyanates during the polymerization. After bulk polymerization, HG(*x*) showed no absorption near 2270 cm^−1^, which indicates that the isocyanate was completely reacted with other units to form urethane or urea linkages. Additionally, the absorption peak of the carbon-oxygen double bond from the urethane or urea linkages appearing at a 1682 cm^−1^ absorption peak also proved the reaction went to completion.

### 3.2. Mechanical and Transparency Properties of Thermoplastic Polyurethane Linked via Host-Guest Interactions

**Figure 3:**
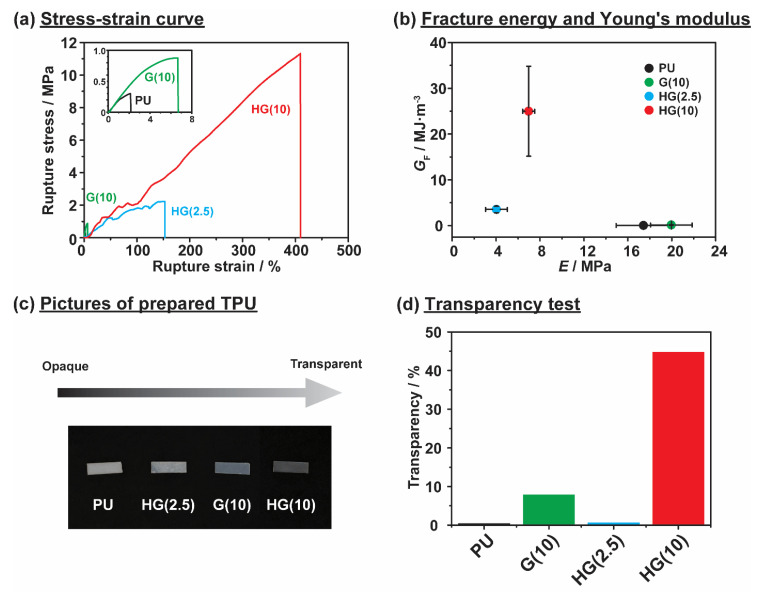
(**a**) Stress strain curve and (**b**) fracture energy (*G*_F_) with Young’s modulus (*E*) of all the prepared TPUs. (**c**) Pictures of all the prepared TPUs. (**d**) UV-Vis transmission in the visible region (λ = 550 nm) to determine the transparencies of the prepared TPUs. The prepared TPUs are host-guest TPU (HG(*x*)), linear TPU (PU), and guest TPU (G(*x*)). The proportions of host and guest units in the TPU derivatives are indicated by “*x*” (HG(*x*) and G(*x*)), which represents the mol% of the PMeAmβCD and AdAm units.

We investigated the mechanical properties of the prepared TPUs by using tensile tests. The stress and strain curves between HG(*x*) and other reference samples while stretching at a tensile speed of 10 mm·min^−1^ are compared in Figure 3a. However, since mechanical properties also correlated with molecular weight [55], we also measured all prepared TPU samples’ molecular weight, as shown in Table S4. These TPU samples with soft segments and linear isocyanates have comparative molecular weight with soft segments from reported research [56]. HG(10) has a large PDI, due to possible dissociation during GPC measurements that used DMSO as eluent. However, it is also common for step-growth polymerization to have a PDI of around 2 [57].

The small number of HG units attached to the TPU already improved its rupture stress. HG(2.5) showed a rupture stress of 2.2 MPa, while that of PU, a non-functionalized TEG-based TPU, was just 0.30 MPa. When the content of HG units was increased to 10 mol% as HG(10), the highest rupture stress (11 MPa) was obtained. To confirm importance of HG interactions, reference sample G(10) was prepared. G(10) showed a lower rupture stress than HG(10) with 0.88 MPa. Figure 3b shows the fracture energy (*G*_F_) and Young’s modulus (*E*), calculated from the stress-strain curve. HG(10) showed the highest *G*_F_ (25 MJ·m^−3^), followed by HG(2.5) with 3.5 MJ·m^−3^, and reference samples G(10) and PU showed *G*_F_ values of 0.10 and 0.017 MJ·m^−3^, respectively. Both the rupture stress and fracture energy results showed that HG units as reversible bond can effectively dispersed stress. Additionally, with higher content of HG units in HG(10), the dispersion of stress resulting in higher mechanical properties both in rupture stress and fracture energy. These results indicate that incorporating HG interactions to TPU plays an important role in increasing its mechanical properties [58].

However, for the *E* results, G(10) and PU showed high *E* values of 20 and 17 MPa, respectively. HG(10) and HG(2.5) showed *E* values approximately half that of G(10) and PU values of 7.0 and 4.0 MPa, respectively. These *E* results are correlated with the steric hindrance in TPU structures. HG(10) and HG(2.5) contain bulky CD groups, which caused substantial steric hindrance in the TPU structures, decreasing the toughness of the structure, due to the fewer interactions and greater distances among the TPU structures. HG(2.5) has less steric hindrance due to fewer HG units. On the other hand, G(10) and PU had low steric hindrance, resulting in more intact structures and shorter distances in the TPU structures. These results are consistent with the transparencies of the TPUs.

Figure 3c,d shows pictures and the results of the transparency tests of the TPU samples. The transparency data were obtained in the visible light region (550 nm). PU with a high *E* showed the lowest transparency with 0.45%, due to the lower steric hindrance and resulting in semicrystalline. HG(2.5) showed the lowest *E* and transparency of 0.65%, similar to those of PU. G(10) with a high *E* showed a transparency of 7.9%, which indicates a slight transition from semicrystalline to amorphous. At 45%, HG(10) was the most transparent among the reference samples, because of the bulky CD structures, which caused substantial steric hindrance and irregular packing of the molecular chains, effectively preventing crystallization.

The transparency data were also supported by differential scanning calorimetry (DSC) data (Figure S13). From the DSC curve, PU (*T*_g_ = −46.5 °C), HG(2.5) (*T*_g_ = −37.5 °C), and G(10) (*T*_g_ = −20.6 °C) showed crystallization temperature peaks (Tc values), but HG(10) (*T*_g_ = −5.0 °C) did not show a *T*_c_ peak. The disappearance of the *T*_c_ peak showed that the TPU structure changed from semicrystalline to amorphous, due to the large amount of CD structures, which caused HG(10) to be highly transparent [59].

### 3.3. Fast Recovery of Thermoplastic Polyurethane Linked via Host-Guest Interactions

**Figure 4:**
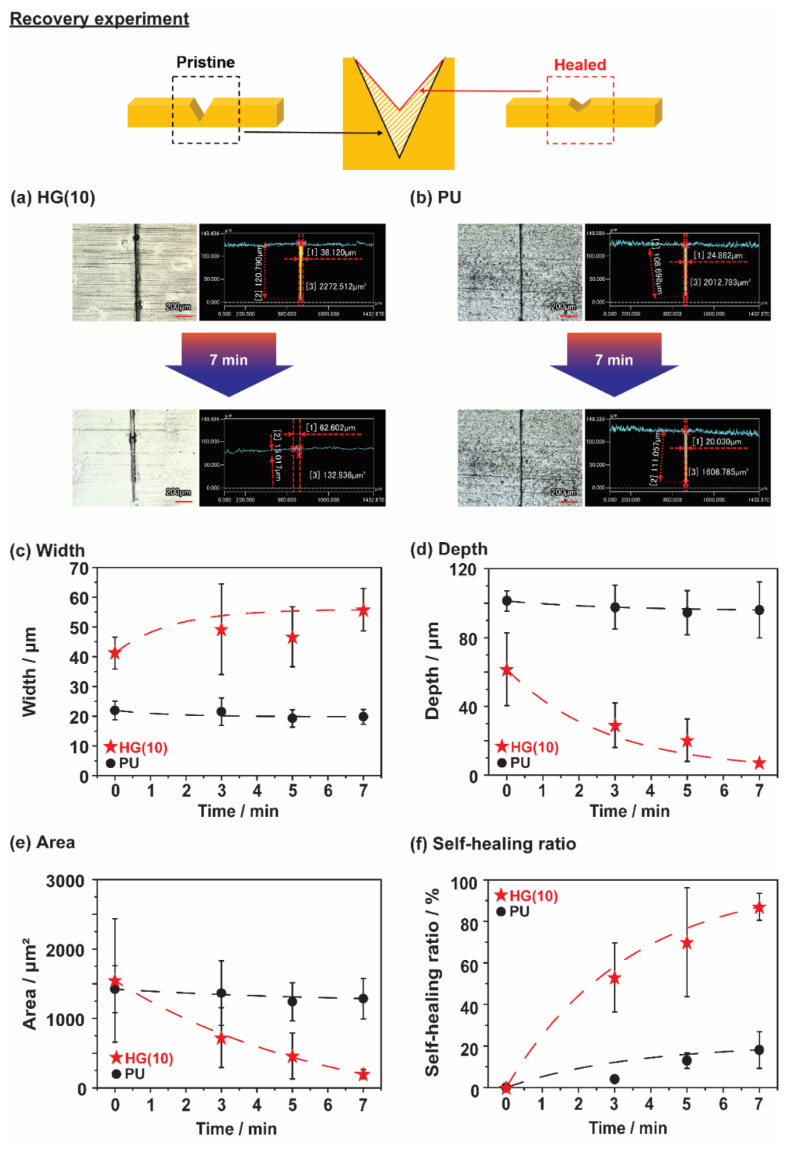
Optical microscopy images of (**a**) HG(10) and (**b**) PU after damaging and placing at 80 °C for 7 min. The scar profiles of HG(10) and PU as (**c**) width, (**d**) depth, and (**e**) area. (**f**) Self-healing ratio of HG(10) and PU calculated from the area.

We investigated the fast recovery of the TPU samples. Figure 4a,b show 3D images and cross-sectional profiles of HG(10) and PU by confocal laser microscopy. The results of other TPU samples (G(10) and H(2.5)) are provided in Supporting Information Figures S14–S17. All the TPU samples were damaged using a razor blade to form a scar at a depth of approximately 140 μm. The samples with scar were heated for a short period in an oven at 80 °C. After 7 min, HG(10) showed disappearance of the scar, whereas the scar remained in PU. Supplementary Movie S1 demonstrated the fast recovery of HG(10) at 80 °C.

Figure 4c–f provide more detail about the scar profile versus time. Figure 4c shows the scar width profiles for both HG(10) and PU. The scar in HG(10) was wider after 7 min (from 41 to 55 μm), while that of PU became narrower after 7 min (from 21 to 19 μm). Figure 4d shows clear trend in the depth profiles. PU showed a slight depth difference (from 101 to 96 μm), but HG(10) showed a significant exponential decrease in depth (from 61 to 7 μm), which means that the damaged HG(10) recovered. The width profile is inversely proportional to the depth and area profiles. This result is consistent with a report by Urban et al., about the curvature of scar surfaces [60,61]. The scar surface begins with small curvature (small width with large depth). At this point, the self-healing is rapid, and it slows as the curvature grows larger (a large width with a shallow depth), or the scar is relatively flat compared to the bottom of the scar.

Based on width and depth, the cross-sectional area (*A*) of the scar was also evaluated to calculate the recovery ratio (Figure 4e,f). The recovery ratio was determined with the following equation:

(1) $$\mathrm{Recovery}\;\mathrm{ratio}=\frac{A_{0}-A_{t}}{A_{0}}\times 100\%$$

where *A*_0_ is the cross-sectional area of the scar on the TPU sample, and *A*_t_ is the cross-sectional area of the scar on the TPU sample after *t* minutes of heating in an oven. Both samples, HG(10) and PU showed similar initial *A* values of 1500 and 1400 μm^2^, respectively. After 7 min, the *A* of PU showed a small recovery to 1200 μm^2^, corresponding to almost 20% recovery, but HG(10) showed a large decrease in *A* to 200 μm^2^, corresponding to 87% recovery. These results show that the reversible bonds from the HG units are necessary for the fast recovery of HG(10).

### 3.4. Self-Healing Properties of Thermoplastic Polyurethane Linked via Host-Guest Interactions

**Figure 5:**
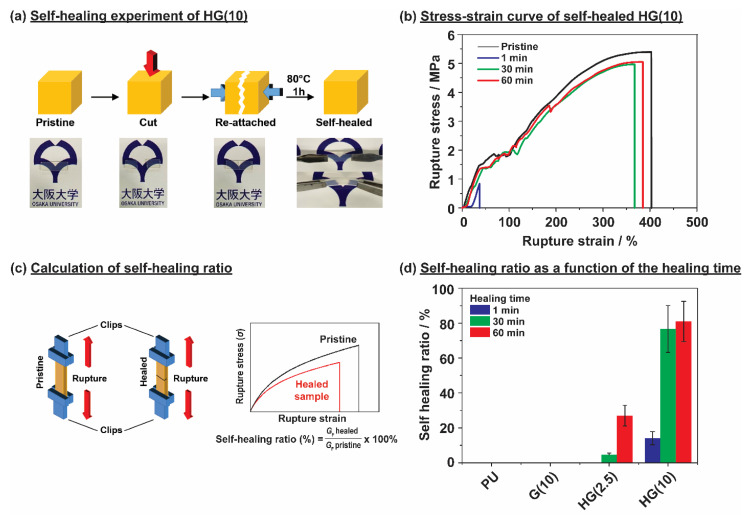
(**a**) Photographs of the self-healing experiment for HG(10). (**b**) Stress-strain curve of self-healed HG(10) at 80 °C after 1 min, 30 min, and 60 min. (**c**) Calculation of the self-healing ratio. (**d**) Self-healing ratios of all the TPUs as a function of time. Calculated from the fracture energy. The prepared TPUs are host-guest TPU (HG(*x*)), linear TPU (PU), and guest TPU (G(*x*)). The proportions of of host and guest units in TPU are indicated by “*x*” (HG(*x*) and G(*x*)), which represents the mol% of the PMeAmβCD and AdAm units.

To further demonstrate the importance of HG units for the fast recovery of TPU, the self-healing properties were evaluated by completely cutting the TPU samples and reattaching them for 1 h at 80 °C. Pristine sample also pre-heated a first at 80 °C to maintain similar condition with self-healed sample. All TPU samples were placed in room temperature for 2 h, before investigating their stress-strain curve. Figure 5a shows the self-healing experiment of HG(10), which showed that, after reattaching for 1 h at 80 °C, HG(10) could be stretched and bent, without any indication of damage. The usage of higher temperature accelerated self-healing demonstration rather than self-healing in ambient temperature. Figure 5b shows the stress-strain curves of HG(10) after different healing times, and these data were acquired for the TPU samples at a tensile velocity of 10 mm·min^−1^. All TPU samples were placed in ambient temperature for just 2 h, therefore, the samples still have some high mobility molecules that did not retain the original condition in the ambient temperature, which caused the lower stress value compared to Figure 3a. The *G*_F_ values calculated from the area under stress-strain curve were used to calculate the self-healing ratio [62].

Figure 5c shows the self-healing ratio calculation. The self-healing ratio was calculated by comparing the *G*_F_ of the self-healed TPU sample (*G*_F_ healed) at *t* min with that of an uncut TPU sample (*G*_F_ pristine). Figure 5d shows the self-healing ratios, and the results of HG(*x*) are very appealing, compared to those of conventional TPU. All the TPU samples were cut and reattached for 1 min, 30 min, and 60 min at 80 °C to explore time dependence of the healing process. All samples were reattached in dry state without any assistance of H_2_O. PU and G(10) showed no healing ability, even after 60 min of reattachment. HG(2.5) showed a 27% self-healing ratio due to the low content of HG units. Interestingly, when the HG content was 10% (HG(10)), the material showed a high self-healing ratio of 80% with low *T*_g_ and no *T*_c_, confirms that the reversible bond from HG units and compatibility with TPU structures in HG(10) are important for preparing self-healing TPU.

### 3.5. Comparison of Thermoplastic Polyurethane Linked by Host-Guest Interactions with Previously Reported Host-Guest Materials

**Figure 6:**
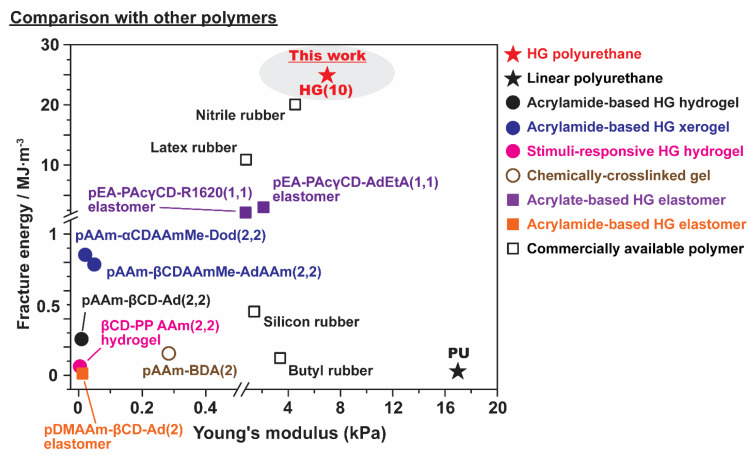
Comparison of the mechanical properties of HG(10) with other polymers. Materials represented by star symbols (★) are the TPUs prepared in this work (red-labeled material is HG(10) and black-labeled material is PU), the materials represented by dots (●) are hydrogels (black-labeled materials from reference [63], blue-labeled materials are from reference [64], and pink-labeled materials are from reference [65]), the materials represented by brown-labeled hollow dots (○) is a chemically cross-linked hydrogels, materials represented by square symbols (■) are elastomers (purple-labeled materials are from reference [49] and orange-labeled materials are from reference [50]), and materials represented by black-labeled hollow square (□) are commercially available polymers.

Figure 6 compares the *G*_F_ and *E* values of HG(10) and PU with those of other polymers, such as HG self-healing materials from our previous works, based on poly(acrylamide) (pAAm and pDMAAm) [63,64,65], and poly(acrylate)s main chain polymers (pEA and pHEA) [49,50], chemically cross-linked gel (pAAm-BDA(2)), and other commercially available polymers (butyl rubber, latex rubber, nitrile rubber, and silicon rubber). HG(10) showed an *E* of approximately 7 MPa, which is lower than that of linear PU but higher than that of any other materials, even nitrile and latex. HG(10) showed the highest *G_F_* among these HG materials, with approximately 25 MJ·m^−3^, and this value is even higher than those of our previously reported HG elastomers (pEA-PAcγCD(1) elastomer and pDMAAm-βCD-Ad(2) elastomer). These results showed that the incorporation of HG interactions into TPU can increase the mechanical properties of TPU.

## 4. Conclusions

In this report, we studied whether the incorporation of HG interactions into urethane can increase the mechanical properties and self-healing ability of the resulting material. Thermoplastic polyurethane derivatives linked by host-guest interactions (HG(*x*)) were successfully prepared by step-growth bulk polymerization from HDI, TEG, and HG interactions between PMeAmβCD and AdAm. HG(10) shows the highest rupture stress and *G*_F_ compared among the tested samples, such as PU and G(10). HG(10) was also shown to be transparent, due to the bulky structures involved in the HG interactions, which changed the TPU structures from semicrystalline to amorphous. The most interesting result was the self-healing properties of HG(*x*). With 10 mol% HG incorporated into the TPU structures, HG(10) showed fast recovery after damaging even after complete cutting. These self-healing properties were closely related to the presence of the HG units. The presence of HG interactions was important for stress dispersion and self-healing properties based on reversible bond formation. We believe that, in the near future, TPU linked via HG interactions may be suitable for industrial applications, due to its toughness and self-healing properties.

## Supplementary Materials

The following are available online at https://www.mdpi.com/2073-4360/12/6/1393/s1, Scheme S1: Synthesis routes for PMeAmβCD from mono-6-*O*-(*p*-toluenesulfonyl)-β-cyclodextrin, Scheme S2: Synthesis of the PMeAmβCD-AdNH_2_ inclusion complex, Scheme S3: Synthesis of the HG(*x*), Scheme S4: Synthesis of the PU, Scheme S5: Synthesis of the G(*x*), Table S1: Feed ratios for HG(2.5), HG(10) components, Table S2: Feed ratios for PU components, Table S3: Feed ratios for G(10) components, Figure S1: 400 MHz ^1^H-NMR spectrum of PMeAmβCD in DMSO-*d_6_*, Figure S2: 100 MHz ^13^C-NMR spectrum of PMeAmβCD in DMSO-*d*_6_, Figure S3: MALDI TOF mass spectrum of PMeAmβCD, Figure S4: 500 MHz ^1^H-NMR spectrum of the host-guest inclusion complex of PMeAmβCD and AdAm in DMSO-*d*_6_, Figure S5: MALDI-TOF mass spectrum of the host-guest inclusion complex of PMeAmβCD and AdAm, Figure S6: ^1^H-^1^H 2D ROESY NMR spectrum for the host-guest inclusion complex of PMeAmβCD and AdAm in D_2_O, Figure S7: 400 MHz ^1^H-NMR spectrum of HG(10) in DMSO-*d_6_*, Figure S8: 400 MHz ^1^H-NMR spectrum of PU in DMSO-*d_6_*, Figure S9: 400 MHz ^1^H-NMR spectrum of G(*x*) in DMSO-*d*_6_, Figure S10: ATR-FTIR spectra for HG(2.5) and HG(10), Figure S11: ATR-FTIR spectra for PU, Figure S12: ATR-FTIR spectra for G(10), Figure S13: DSC curve of TPU materials, Figure S14: The optical microscopy images of the initial damaged G(10) sample and after 7 min of heating by confocal laser microscopy, Figure S15: The scar profiles of G(10) as (a) width, (b) depth, and (c) area. (d) Self-healing ratio of G(10) calculated from the area, Figure S16: The optical microscopy images of the initial damaged HG(2.5) sample and after 7 min of heating by confocal laser microscopy, Figure S17: The scar profiles of HG2.5 as (a) width, (b) depth, and (c) area. (d) Self-healing ratio of HG(2.5) calculated from the area, Video S1: Self-healing test of HG(10) at 80 °C after damaging with razor blade.
