# Supplementary material for: Self-Healing Thermoplastic Polyurethane Linked via Host-Guest Interactions
Source: Polymers (Basel). 2020 Jun 22;12(6):1393. doi: 10.3390/polym12061393 (PMC7361818; doi:10.3390/polym12061393)
Supplement: Supplementary file 1 [file polymers-12-01393-s001.zip › polymers-816882-supplementary.docx]

Supporting Information

Self-healing Thermoplastic Polyurethane Linked via Host-Guest Interactions

*Changming Jin ^1^, Garry Sinawang ^1,2^, Motofumi Osaki ^1,2^, Yongtai Zheng ^2^,*

*Hiroyasu Yamaguchi ^1,2^, Akira Harada ^3,^*, and Yoshinori Takashima ^1,2,4,^**

* Correspondence: [harada@chem.sci.osaka-u.ac.jp](mailto:harada@chem.sci.osaka-u.ac.jp); [takasima@chem.sci.osaka-u.ac.jp](mailto:takasima@chem.sci.osaka-u.ac.jp);

Tel.: +81-6-6850-5447 (Y.T.)

**CONTENTS**

1. Preparation of permethylated 6-monoamino-β-cyclodextrin (PMeAmβCD)
2. Preparation of PMeAmβCD-AdAm inclusion complex
3. Preparation of TPUs
4. FTIR characterization of TPUs
5. Molecular weight of TPUs
6. DSC curves of TPUs
7. Laser microscope images and scar profile

**1. Preparation of the permethylated 6-monoamino-β-cyclodextrin (PMeAmβCD)**


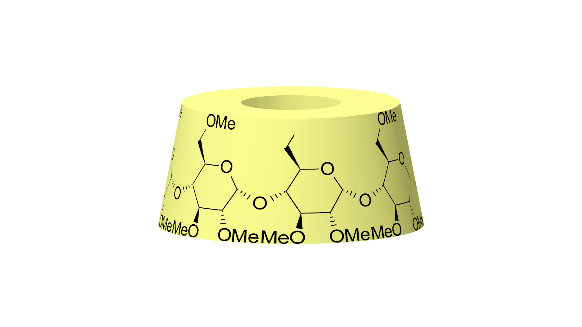


N_3_

**
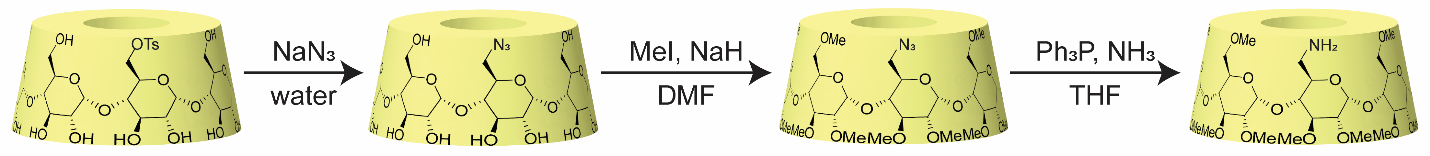
**

**Scheme S1.** Synthesis routes for PMeAmβCD.

The 6-mono-N_3_-βCDOMe was prepared from mono-6-O-(*p*-toluenesulfonyl)-β-cyclodextrin following the procedures of Hocquelet et al.[1] 6-mono-N_3_-βCDOMe (106 g) were dissolved in THF, PH_3_P (41.5 g) and NH_3_·H_2_O (318 ml) was added thereto, and the mixture was stirred at room temperature 18 h. After the solvent was distilled off, performed multiple extractions (1.20 L of H_2_O × 2, 1.00 L brine). After dehydration, the residue was purified by column chromatography (MeOH:CH_2_Cl_2_ = 1:9) to give yellow PMeAmβCD solids (58.0 g).

**^1^H NMR (400 MHz, DMSO-*d*_6_):** δ = 5.15-5.01 (m, 7H, C^1^*H*), 3.75-3.65 (m, 12H, MeO-C^6^*H*_2_), 3.53-3.41 (m, 7H, C^5^*H*), 3.40-3.38 (m, 7H, C^3^*H*), 3.25-3.23 (m, 7H, C^2^*H*), 3.53-3.18 (m, 60H, -OC*H*_3_), 3.09-3.01 (m, 7H, C^4^*H*), 2.90 (m, 2H, N*H*_2_-C^6^H_2_).

**^13^C NMR (100 MHz, DMSO-*d*_6_):** δ = 98.31 (C^1^), 82.13 (C^3^), 81.75 (C^2^), 80.21 (C^4^), 71.62 (C^6^-OCH_3_), 70.88 (C^5^), 61.27 (C^6^-NH_2_), 58.85-58.34 (CH_3_O).


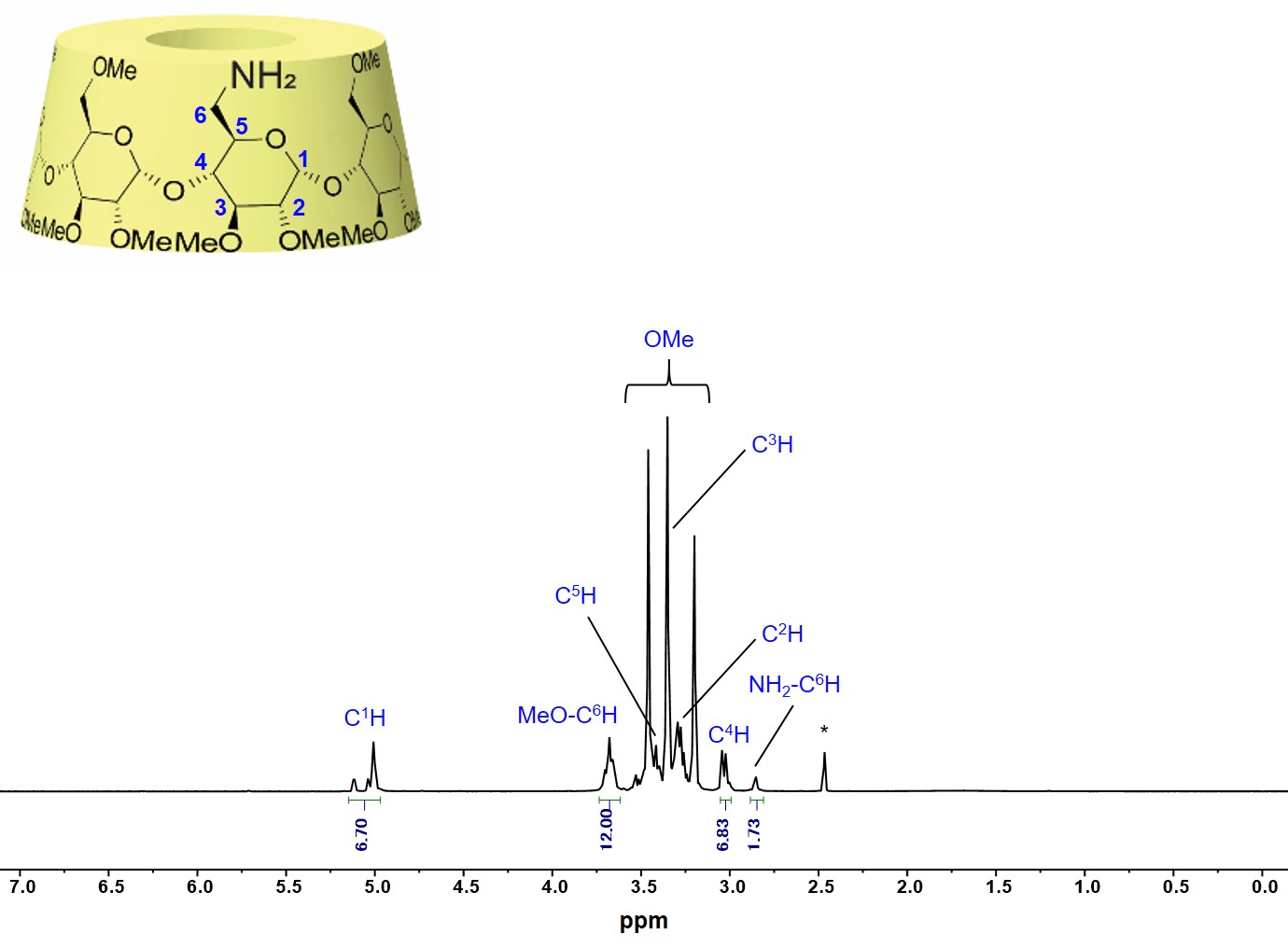


**Figure S1.** 400 MHz ^1^H-NMR spectrum of PMeAmβCD in DMSO-*d*_6_. * shows residual proton of the deuterated solvent.


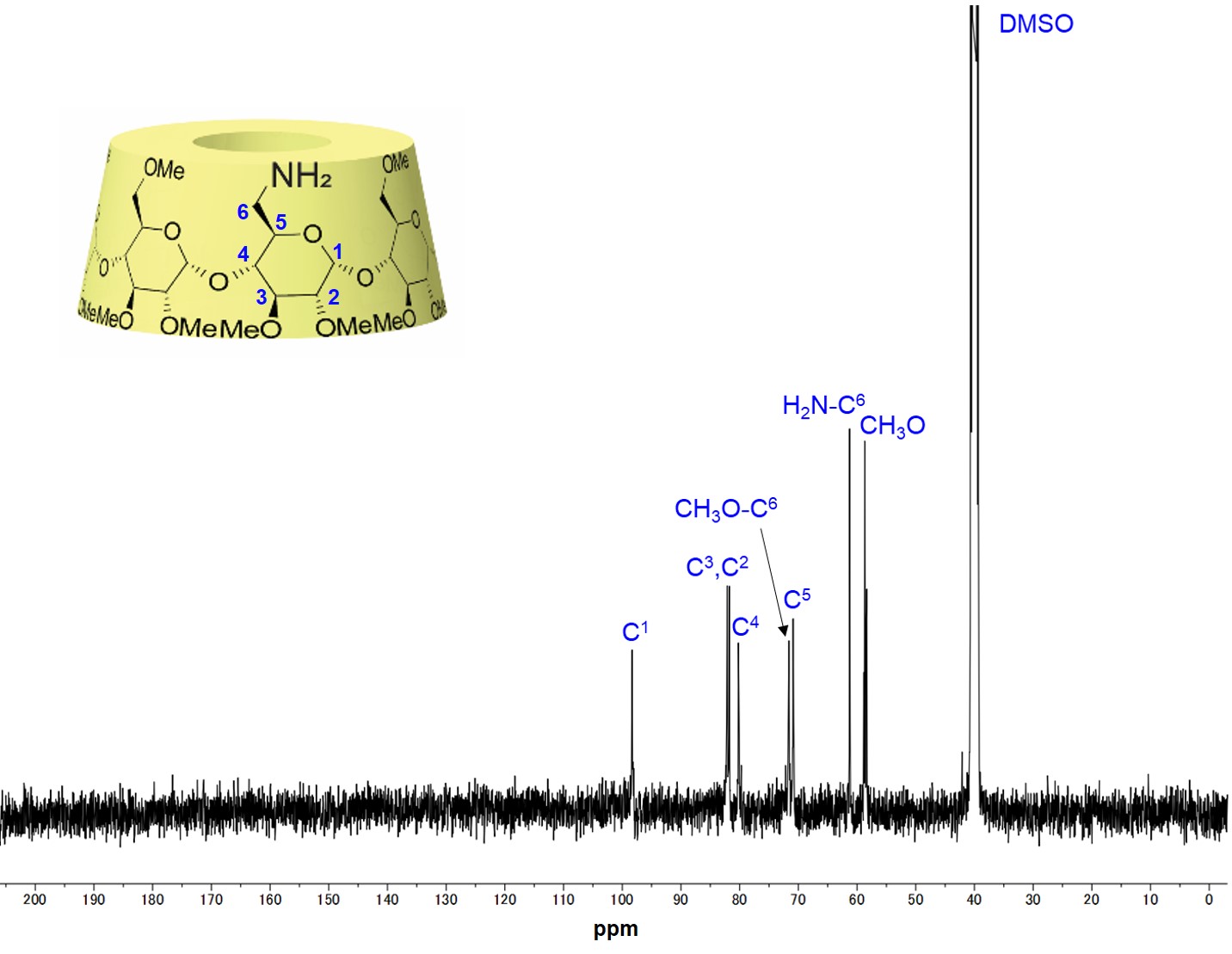


**Figure S2.** 100 MHz ^13^C-NMR spectrum of PMeAmβCD in DMSO-*d*_6_.

**MALDI TOF MS:**

Calcd. *m*/*z* for PMeAmβCD: [M]^+^ = 1413.70, [M+H]^+^ = 1414.71, [M+Na]^+^ = 1436.70.

Found: [M+H]^+^ = 1414.29, [M+Na]^+^ = 1436.27.

**Figure S3.** MALDI TOF mass spectrum of PMeAmβCD.

**2. Preparation of the PMeAmβCD-AdAm inclusion complex**


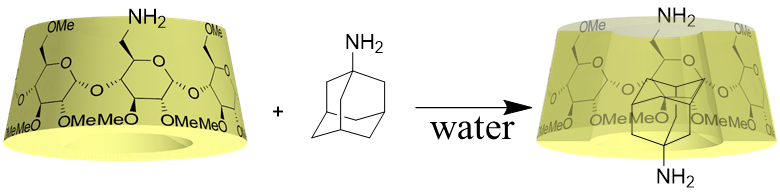

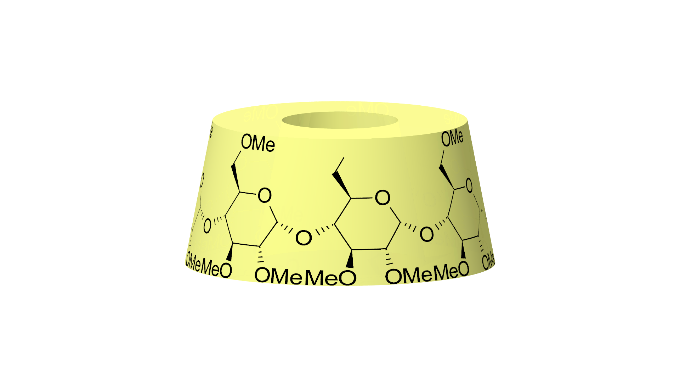


NH_2_


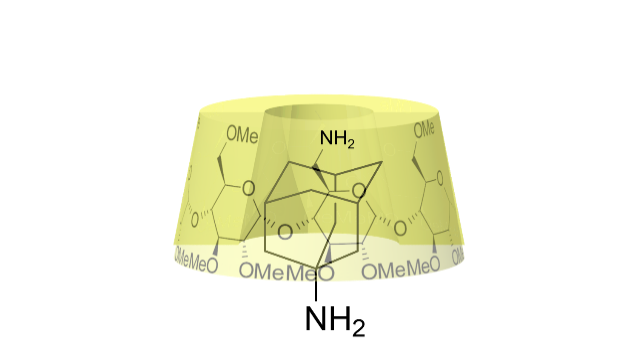


**Scheme S2.** Synthesis of the PMeAmβCD-AdNH_2_ inclusion complex.

Put 14.15 g of NH_2_βCDOMe and 200 mL of H_2_O in a 500 mL Erlenmeyer flask, after completely dissolve, add 1.517 g of 1-adamantamine with stirring, and heat to 90 °C for 1 hour. Thereafter, the temperature was lowered to room temperature, the products were filtered, then placed in a refrigerator overnight. Finally, the products obtained by freeze drying.

**^1^H NMR (400 MHz, DMSO-*d*_6_):** δ = 5.15-5.01 (m, 7H, C^1^*H*), 3.75-3.65 (m, 12H, MeO-C^6^*H*_2_), 3.53-3.41 (m, 7H, C^5^*H*), 3.40-3.38 (m, 7H, C^3^*H*), 3.25-3.23 (m, 7H, C^2^*H*), 3.53-3.18 (m, 60H, -OCH_3_, 3.09-3.01 (m, 7H, C^4^*H*), 2.90 (m, 2H, N*H*_2_-C^6^H_2_), 1.96 (s, 3H, *H*^a^ in Ad), 1.58-1.51 (t, 6H, *H*^b^ in Ad), 1.48-1.47 (d, 6H, *H*^c^ in Ad).

**
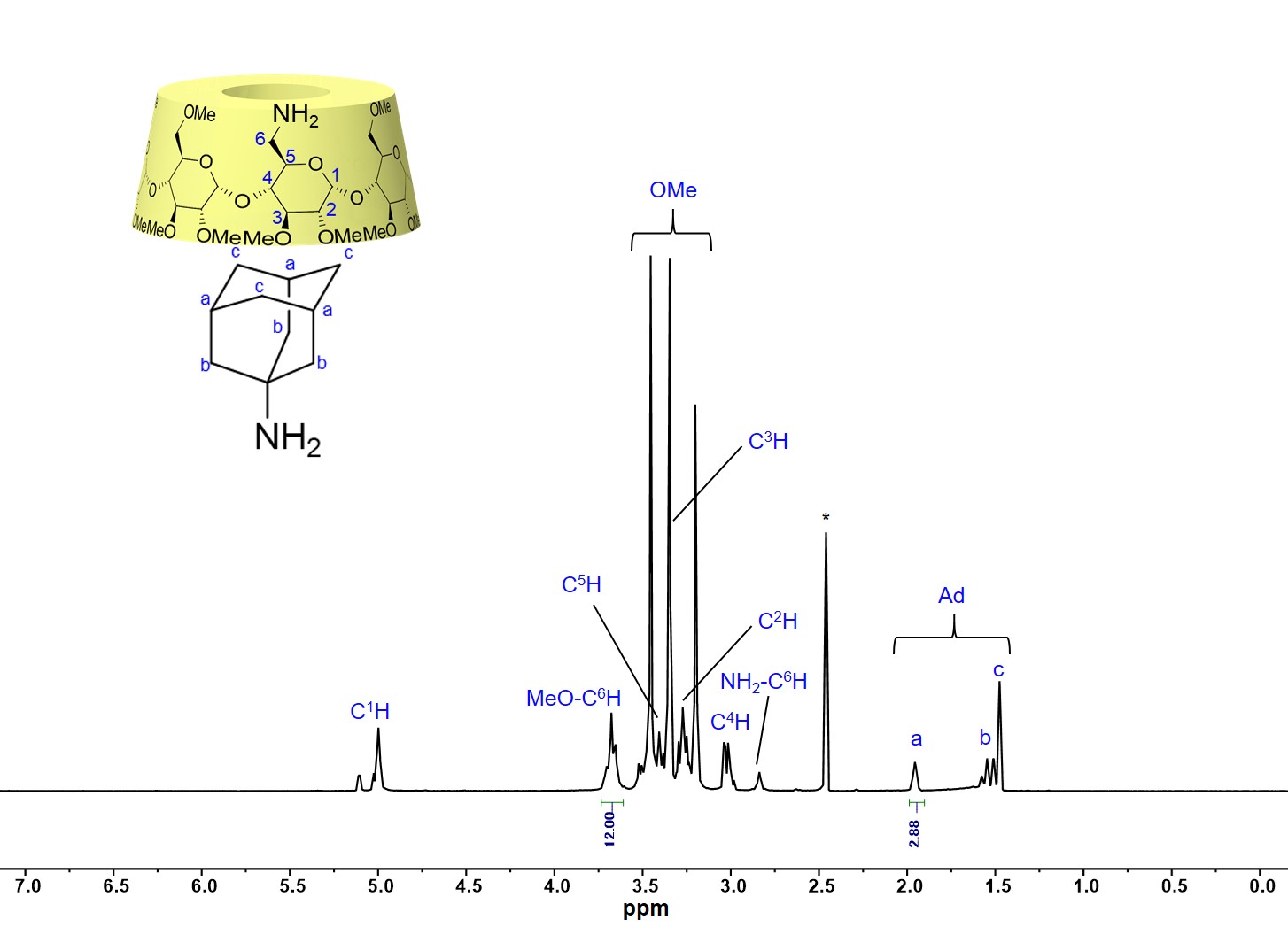
**

**Figure S4.** 500 MHz ^1^H-NMR spectrum of the host-guest inclusion complex of PMeAmβCD and AdAm in DMSO-*d*_6_. * shows residual proton of the deuterated solvent.

**MALDI TOF MS:**

Calcd. for complex of PMeAmβCD and AdAm: *m*/*z* = 1564.95,

Found: [M+H]^+^ = 1566.27, [M+Na]^+^ = 1588.21.

**Figure S5.** MALDI-TOF mass spectrum of the host-guest inclusion complex of PMeAmβCD and AdAm.


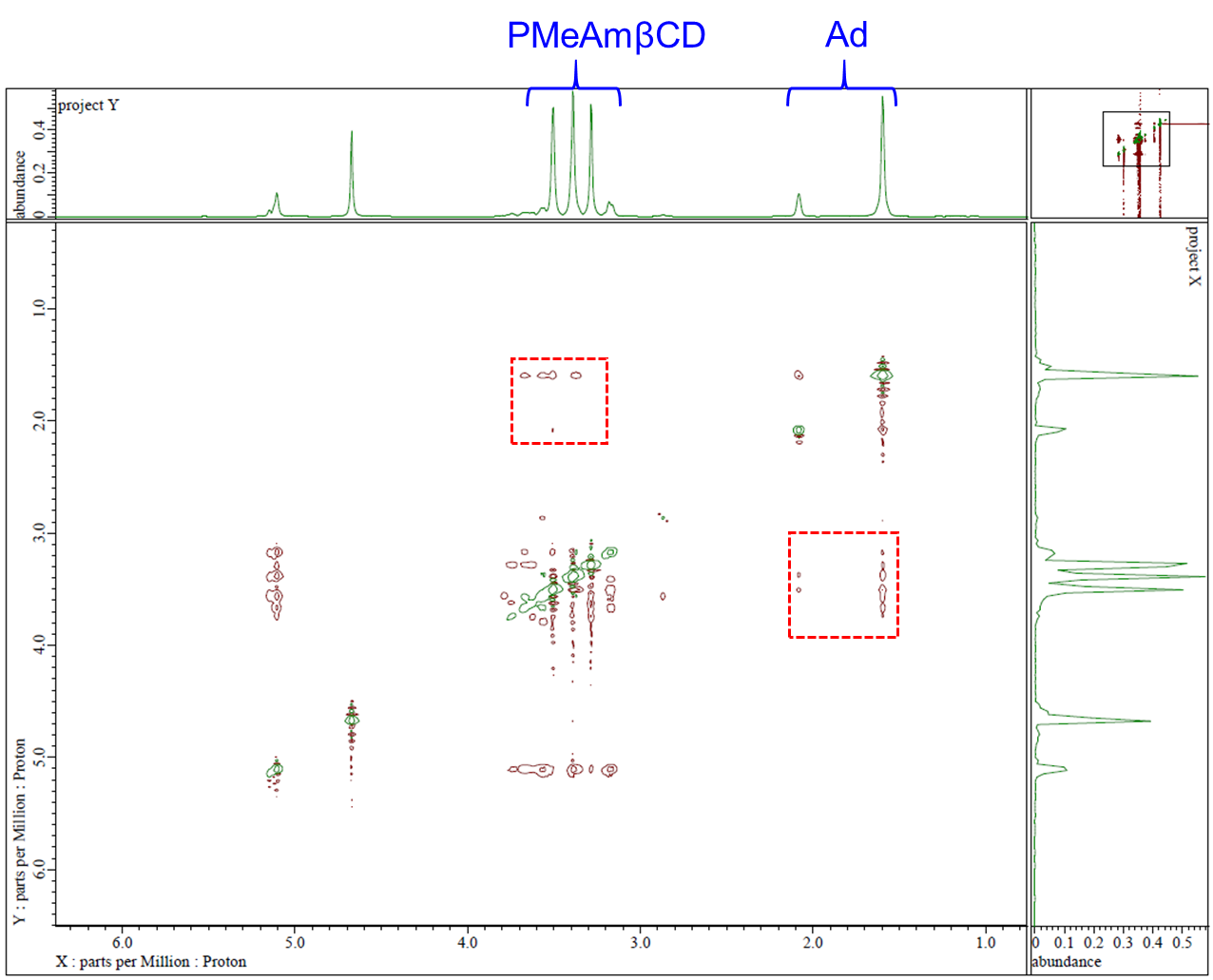


**Figure S6.** ^1^H-^1^H 2D ROESY NMR spectrum for the host-guest inclusion complex of PMeAmβCD and AdAm in D_2_O.

**3. Preparation of thermoplastic polyurethanes (TPUs)**

**Preparation of the** **host-guest polyurethane [HG(*x*)]**

**
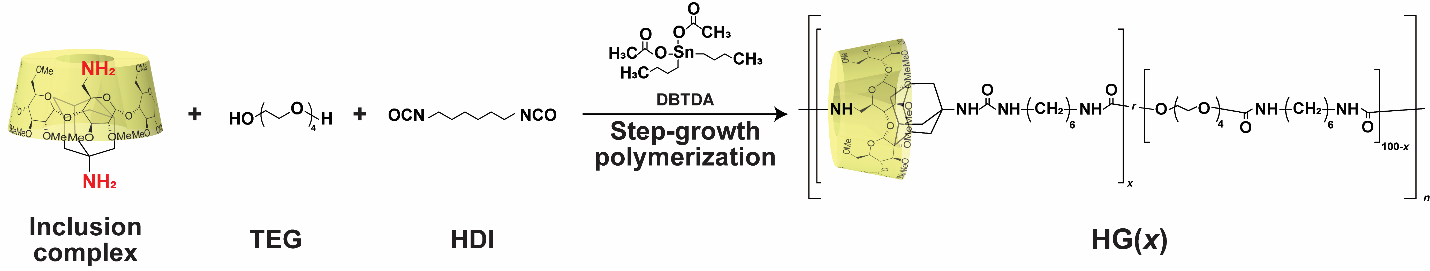
**

**Scheme S3.** Synthesis of the HG(*x*).

Predetermined amounts of HG and TEG were mixed together and vacuum dried at 60 °C for 5 h to remove residual moisture. After cooling down to 4 °C in a refrigerator, HDI and DBTDA were added and the solution was vigorously homogenized. The mixture was rapidly charged into a Teflon mold and allowed to cure at room temperature for 18 h and 70 °C 6 h.

**Table S1.** Feed ratios for HG(2.5), HG(10) components.

| **Samples** | **HG / g** | **TEG / g** | **HDI / g** | **DBTDA / mg** |
| --- | --- | --- | --- | --- |
| HG(2.5) | 0.247 | 1.19 | 1.06 | 1.67 |
| HG(10) | 0.783 | 0.875 | 0.842 | 1.67 |

**^1^H NMR (400 MHz, DMSO-*d*_6_):** δ = 7.17-7.13 (CO_2_-N*H*), 5.69-5.66 (CO-N*H*), 5.55-5.38 (Ad-N*H*), 5.15-5.01 (*H*^1^ in CD), 4.55 (O*H*), 3.99(*H*^f^ in PU), 3.75-3.65 (MeO-C^6^*H_2_* in CD), 3.50 (*H*^g^ in PU), 3.46 (*H*^e^ in PU), 3.53-3.41 (C^5^*H* in CD), 3.40-3.38 (C^3^*H* in CD), 3.25-3.23 (C^2^*H* in CD), 3.53-3.18 (-OCH_3_), 3.09-3.01 (C^4^*H* in CD), 2.92-2.87 (NH-C^6^*H*_2_ in CD; *H*^b^ in PU), 1.96 (*H*^a^ in Ad), 1.58-1.51 (*H*^b^ in Ad), 1.48-1.47 (*H*^c^ in Ad), 1.33-1.26 (*H*^d^ in PU), 1.17(*H*^c^ in PU).


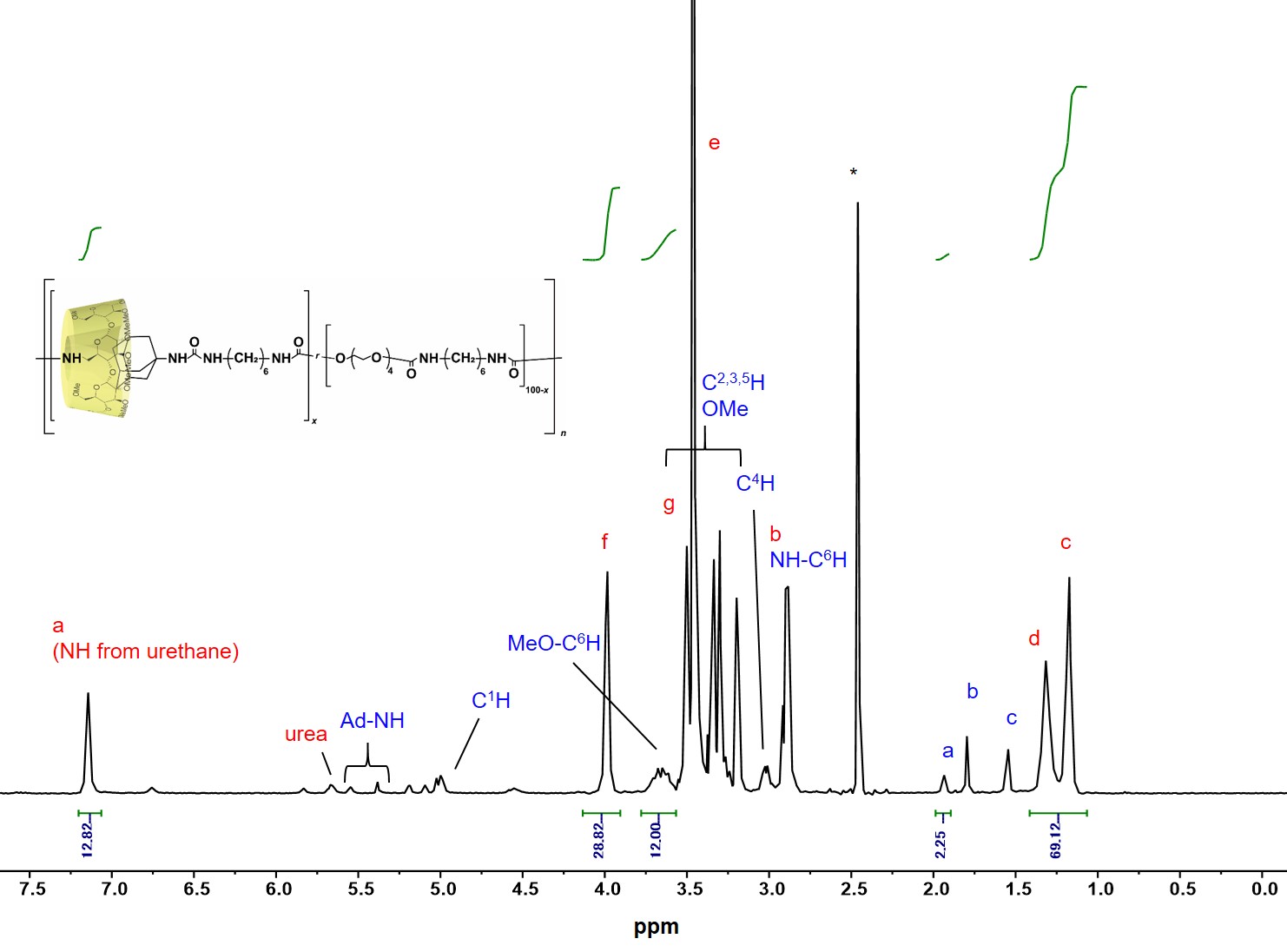


**Figure S7.** 400 MHz ^1^H-NMR spectrum of HG(10) in DMSO-*d*_6_. * shows residual proton of the deuterated solvent.

**Preparation of the linear polyurethane (PU)**

**
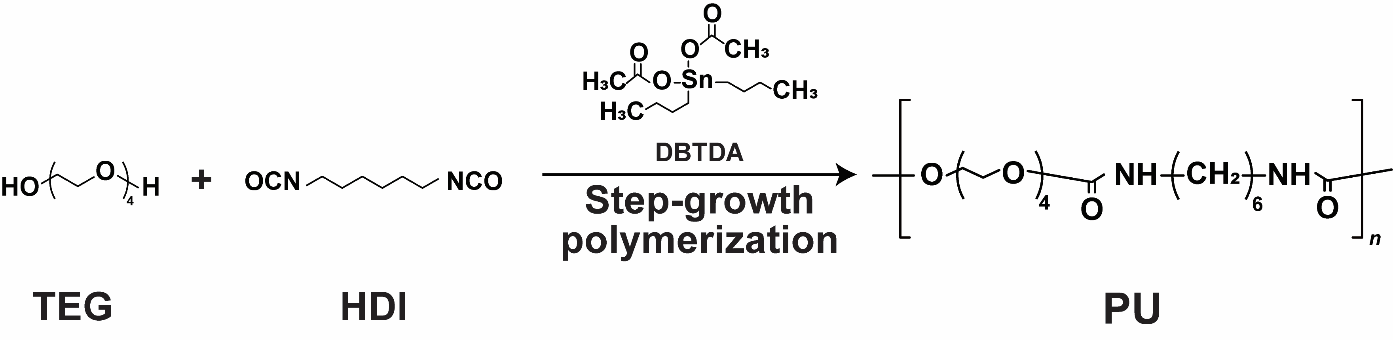
**

**Scheme S4.** Synthesis of the PU.

TEG were vacuum dried at 60 °C for 5 h to remove residual moisture. After cooling down to 4 °C in a refrigerator, HDI and DBTDA were added and the solution was vigorously homogenized. The mixture was rapidly charged into a Teflon mold and allowed to cure at room temperature for 18 h and 70 °C 6 h.

**Table S2.** Feed ratios for PU components.

| **Samples** | **TEG / g** | **HDI / g** | **DBTDA / mg** |
| --- | --- | --- | --- |
| PU | 1.34 | 1.16 | 1.67 |

**^1^H NMR (400 MHz, DMSO-*d*_6_):** δ = 7.17-7.13 (CO_2_-N*H*), 5.69-5.66 (CO-N*H*), 4.55 (O*H*), 3.99(*H*^f^ ), 3.50 (*H*^g^), 3.46 (*H*^e^), 2.92-2.87 (*H*^b^), 1.33-1.26 (*H*^d^), 1.17 (*H*^c^).


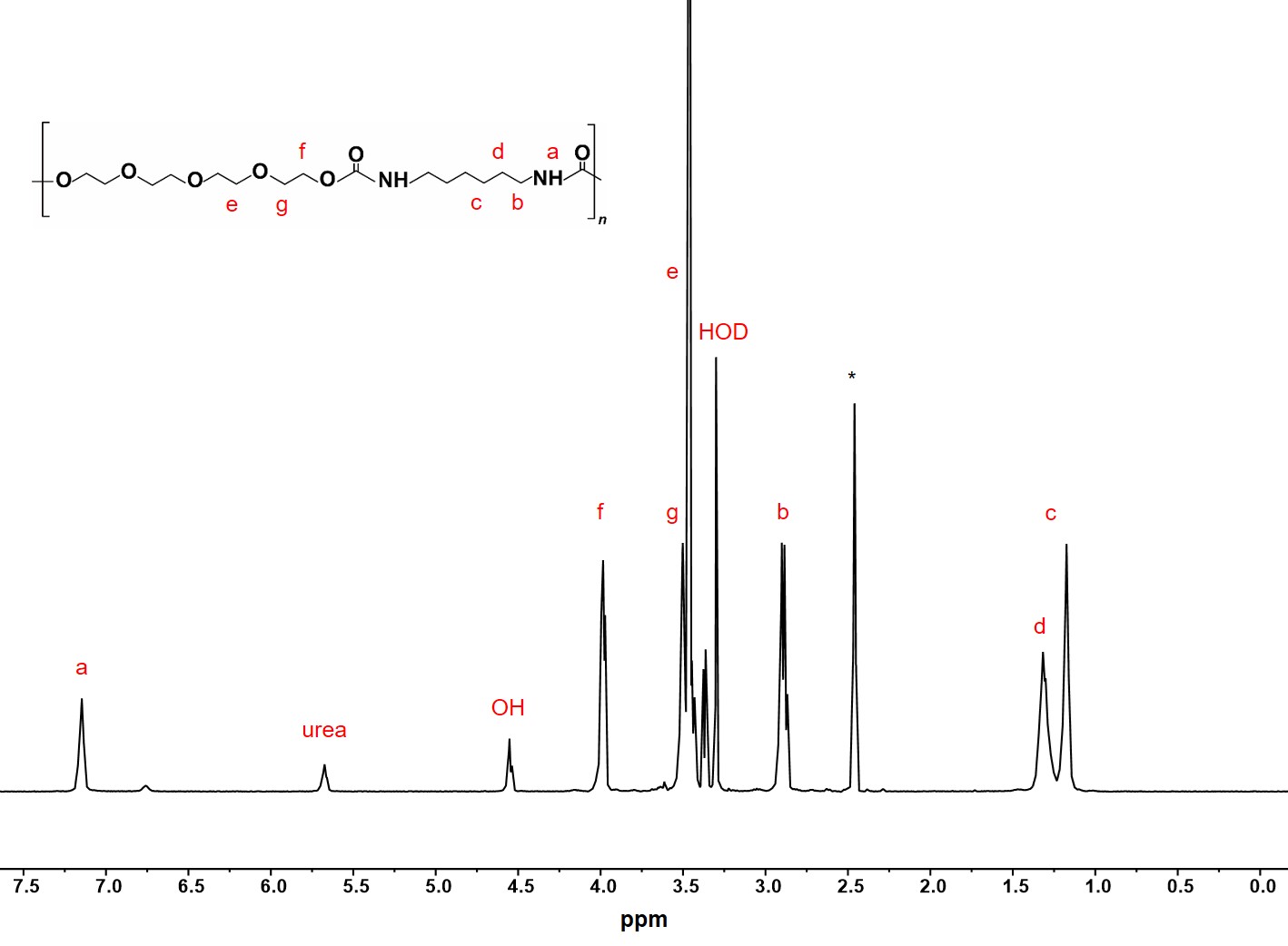


**Figure S8.** 400 MHz ^1^H-NMR spectrum of PU in DMSO-*d*_6_. * shows residual proton of the deuterated solvent.

**Preparation of the guest polyurethane [G(*x*)]**

**
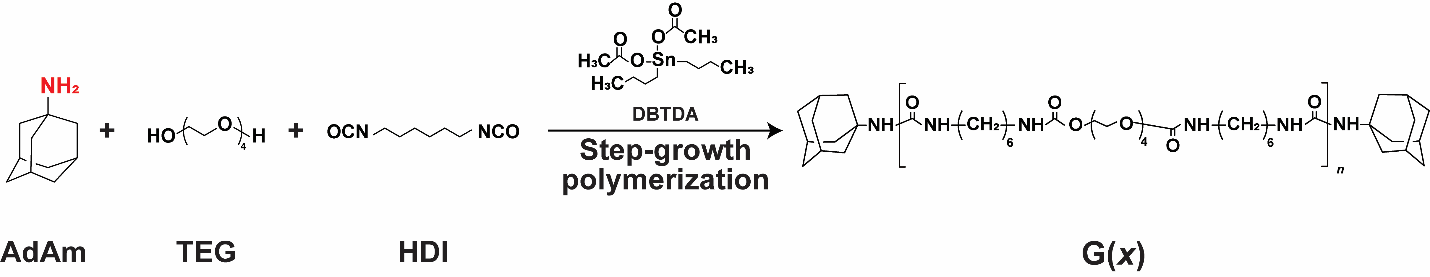
**

**Scheme S5.** Synthesis of the G(*x*)

Predetermined amounts of AdAm and TEG were mixed together and vacuum dried at 60 °C for 5 h to remove residual moisture. After cooling down to 4 °C in a refrigerator, HDI and DBTDA were added and the solution was vigorously homogenized. The mixture was rapidly charged into a Teflon mold and allowed to cure at room temperature for 18 h and 70 °C 6 h.

**Table S3.** Feed ratios for G(10) components.

| **Samples** | **AdAm / g** | **TEG / g** | **HDI / g** | \| **DBTDA / mg** \| \| --- \| |
| --- | --- | --- | --- | --- | --- |
| G(10) | 0.087 | 1.000 | 0.914 | 1.33 |

**^1^H NMR (400 MHz, DMSO-*d*_6_):** δ = 7.17-7.13 (CO_2_-N*H*), 5.69-5.66 (CO-N*H*), 5.55-5.38 (Ad-N*H*), 4.55 (O*H*), 3.99(*H*^f^ in PU), 3.50 (*H*^g^ in PU), 3.46 (*H*^e^ in PU), 2.92-2.87 (*H*^b^ in PU), 1.96 (*H*^a^ in Ad), 1.58-1.51 (*H*^b^ in Ad), 1.48-1.47 (*H*^c^ in Ad), 1.33-1.26 (*H*^d^ in PU), 1.17(*H*^c^ in PU).


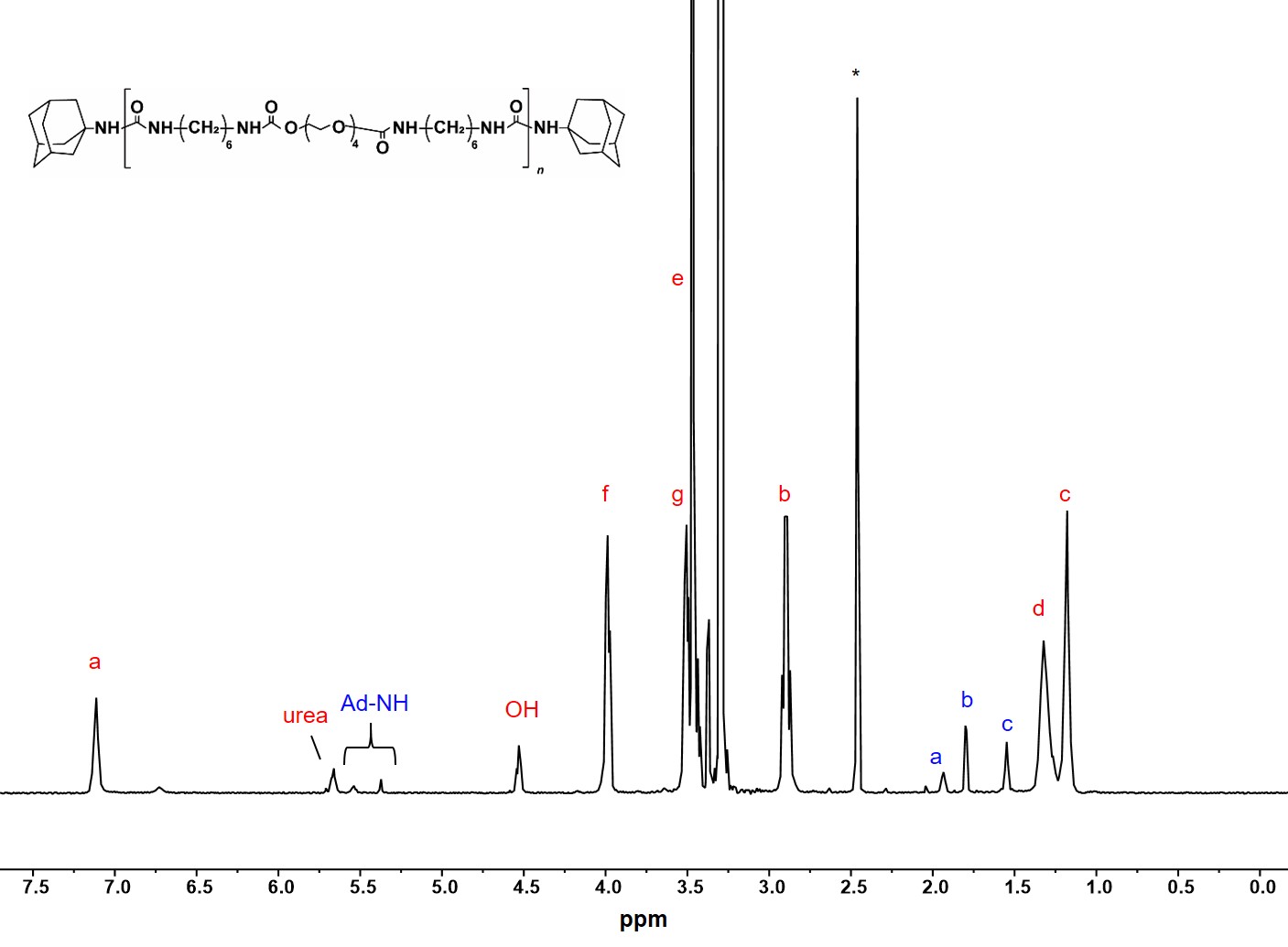


**Figure S9.** 400 MHz ^1^H-NMR spectrum of G(*x*) in DMSO-*d*_6_. * shows residual proton of the deuterated solvent.

**4. FTIR characterization of TPUs**


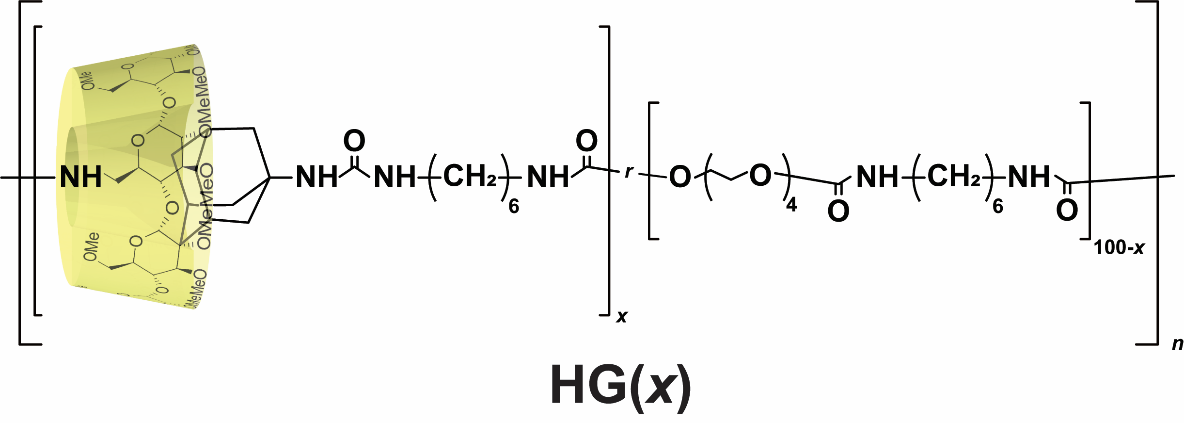


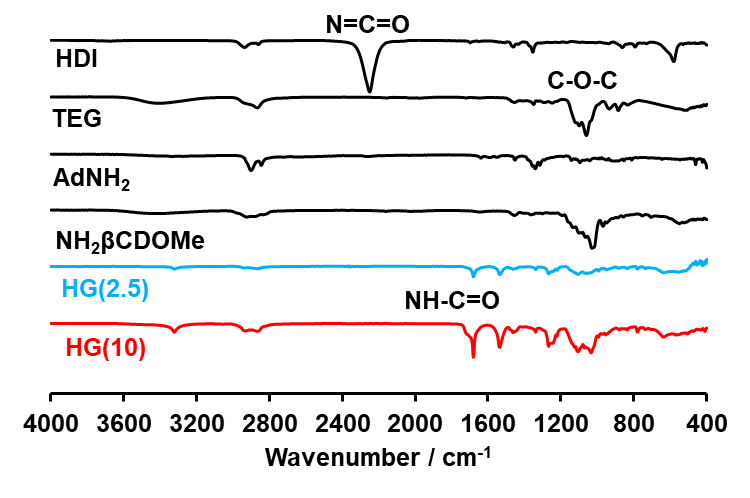


**Figure S10.** ATR-FTIR spectra for HG(2.5) and HG(10). And the ATR-FTIR spectra of HDI, TEG, AdNH_2_ and NH_2_βCDOMe as a reference.


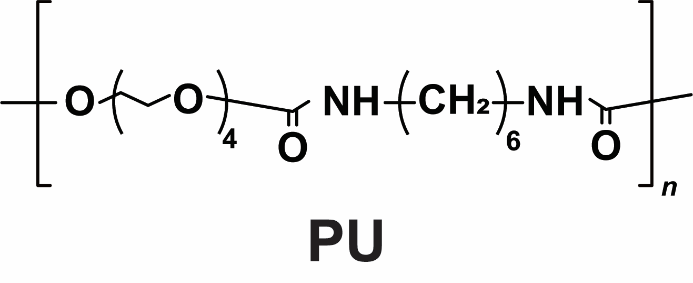


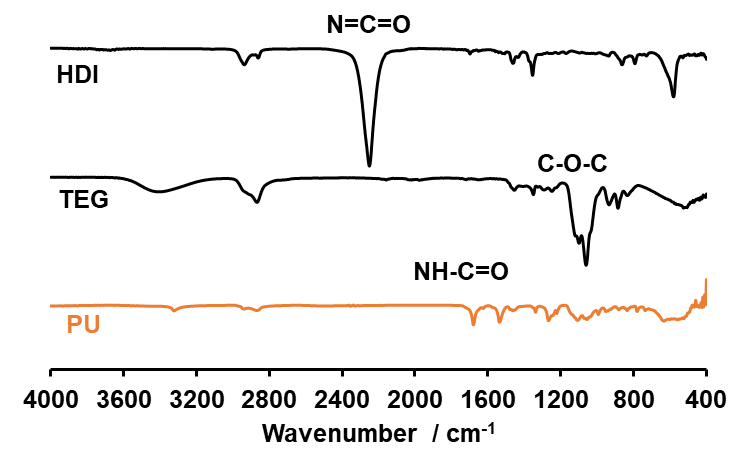


**Figure S11.** ATR-FTIR spectra for PU. And the ATR-FTIR spectra of HDI and TEG as a reference.


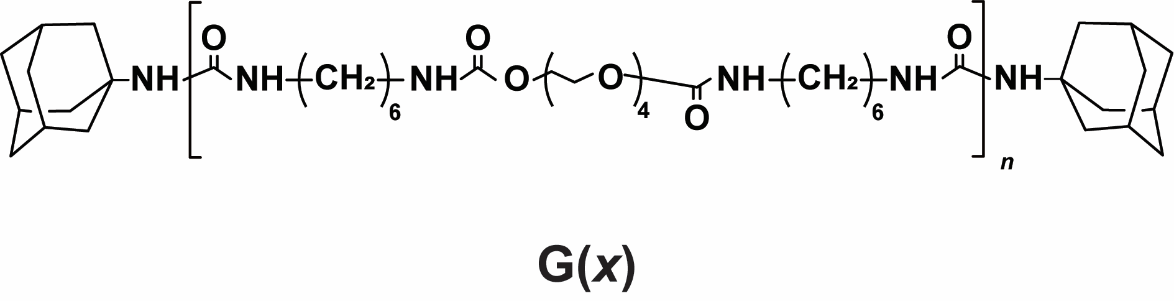


**
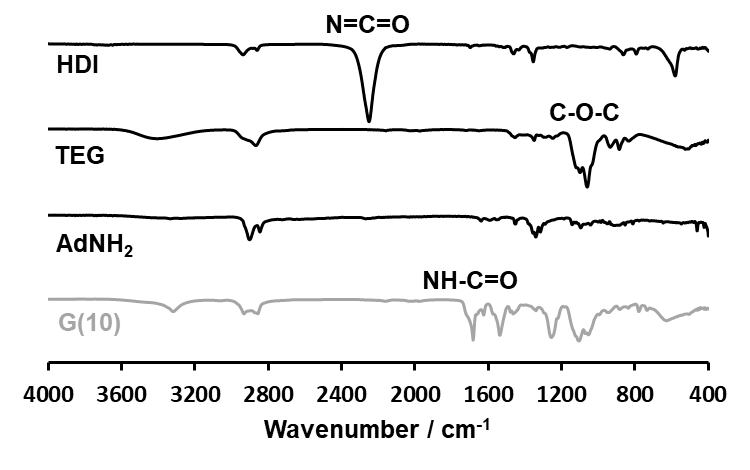
**

**Figure S12.** ATR-FTIR spectra for G(10). And the ATR-FTIR spectra of HDI, TEG, and AdNH_2_ as a reference.

**5. Molecular weight of TPUs**

**Table S4.** Molecular weight of TPU samples

| **TPU samples** | ***M*_n_** | ***M*_w_** | **PDI** |
| --- | --- | --- | --- |
| PU | 2,800 | 4,800 | 1.73 |
| G(10) | 2,400 | 3,800 | 1.61 |
| HG(2.5) | 4,300 | 7,400 | 1.73 |
| HG(10) | 4,500 | 11,800 | 2.62 |

**6. DSC curve of TPUs**

**
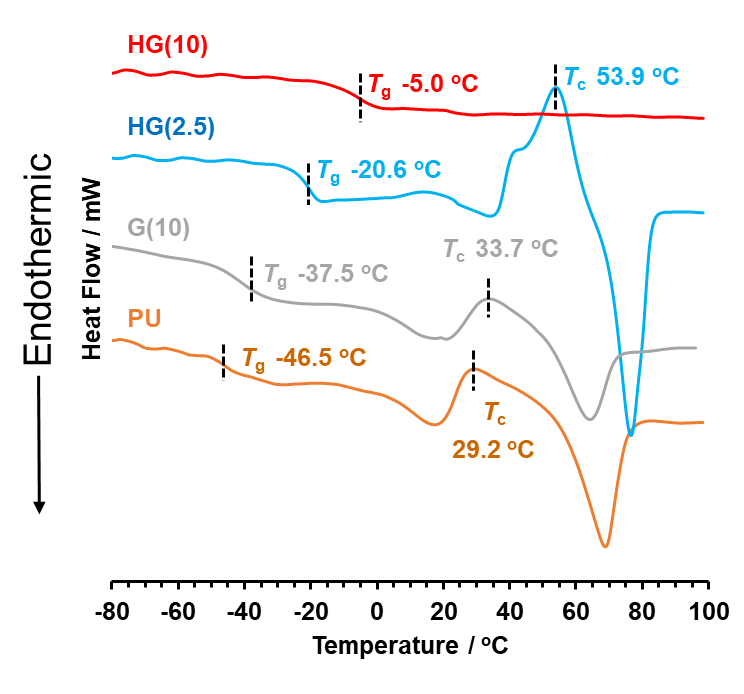
**

**Figure S13.** DSC curve of TPU materials. The temperature range is from -80 °C to 100 °C, the heating rate is 10 °C/min.

**7. Laser microscope images and scratch profile**

**G(10)**


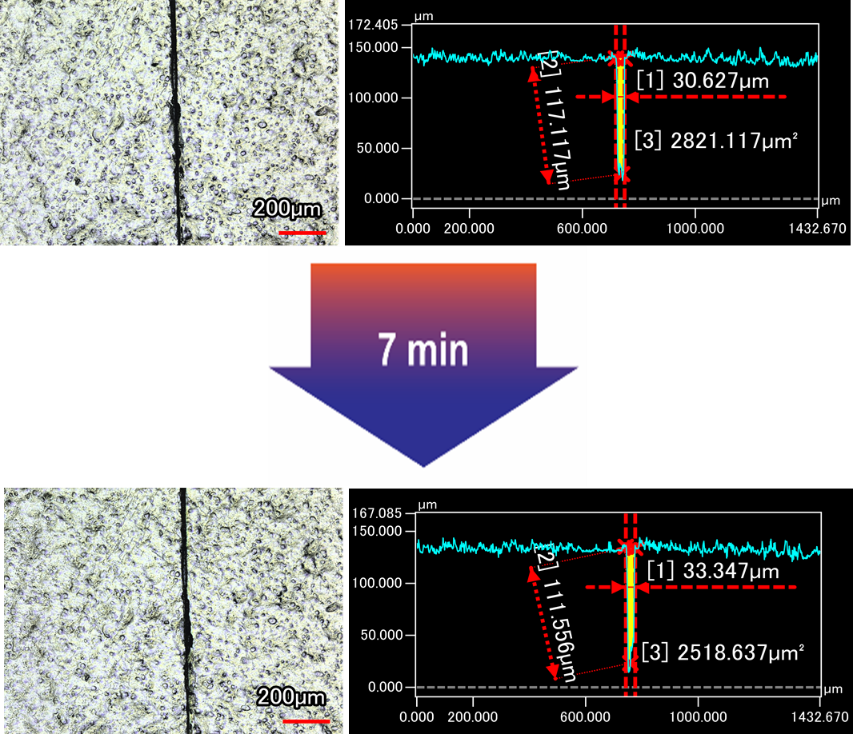


**Figure S14.** The optical microscopy images of the initial damaged G(10) sample and after 7 minutes of heating by confocal LASER microscopy. The sample was placed on the rubber sheet, and the surface of the test sample was cut by knife blade. Then, the sample was placed in the windy oven heated by 80 °C for 7 minutes to recover its cut.


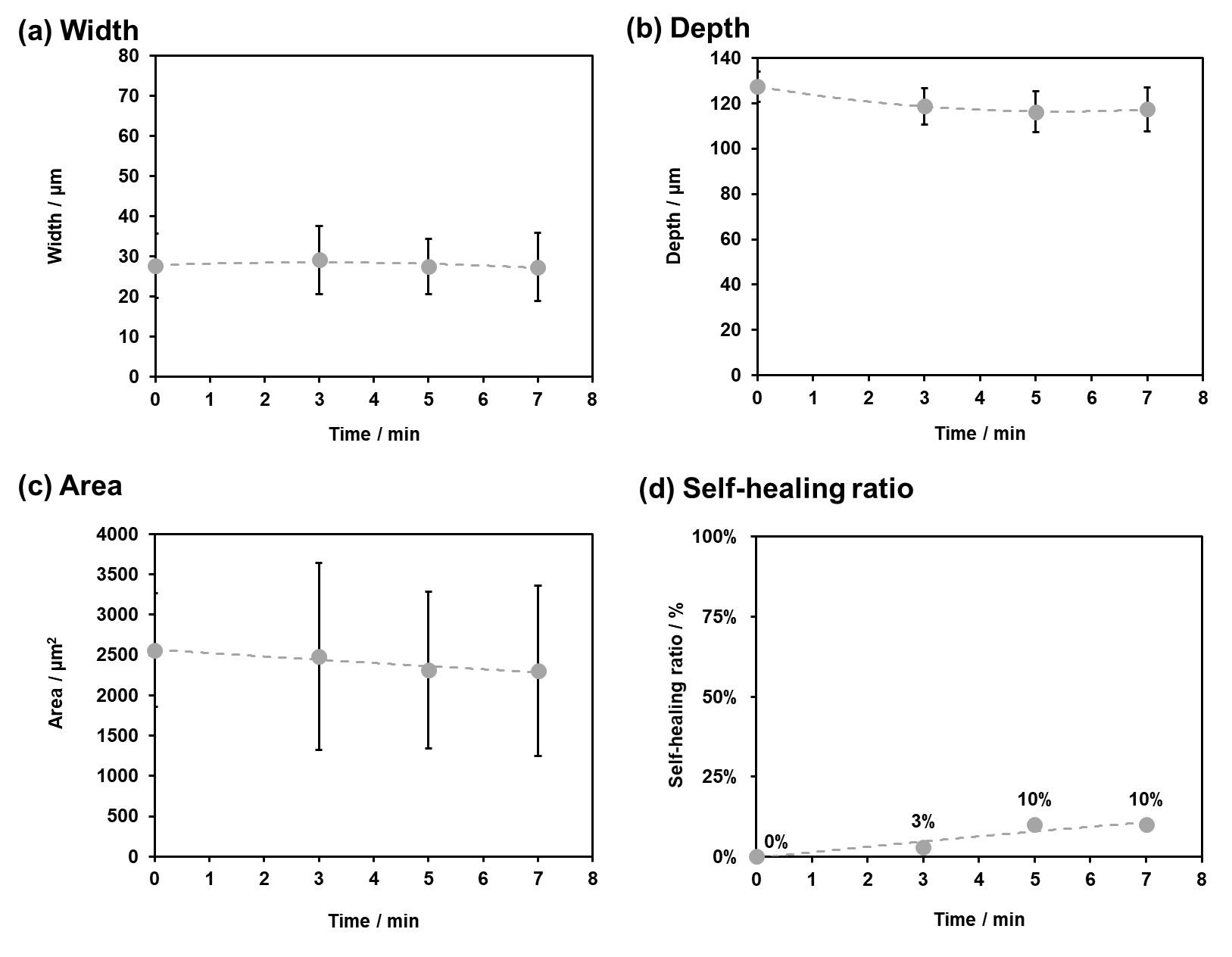


**Figure S15.** The scar profiles of G(10) as (**a**) width, (**b**) depth, and (**c**) area. (**d**) The self-healing ratio of G10 calculated from the area. The error bar indicates sample standard deviation (n = 5).


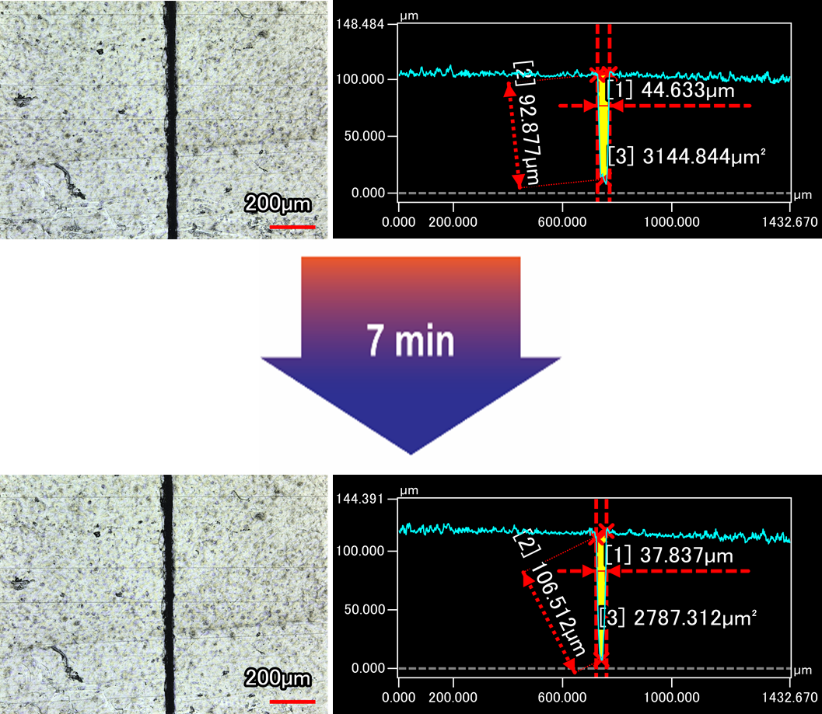
**HG(2.5)**

**Figure S16.** The optical microscopy images of the initial damaged HG(2.5) sample and after 7 minutes of heating by confocal LASER microscopy. The sample was placed on the rubber sheet, and the surface of the test sample was cut by knife blade. Then, the sample was placed in the windy oven heated by 80 °C for 7 minutes to recover its cut.


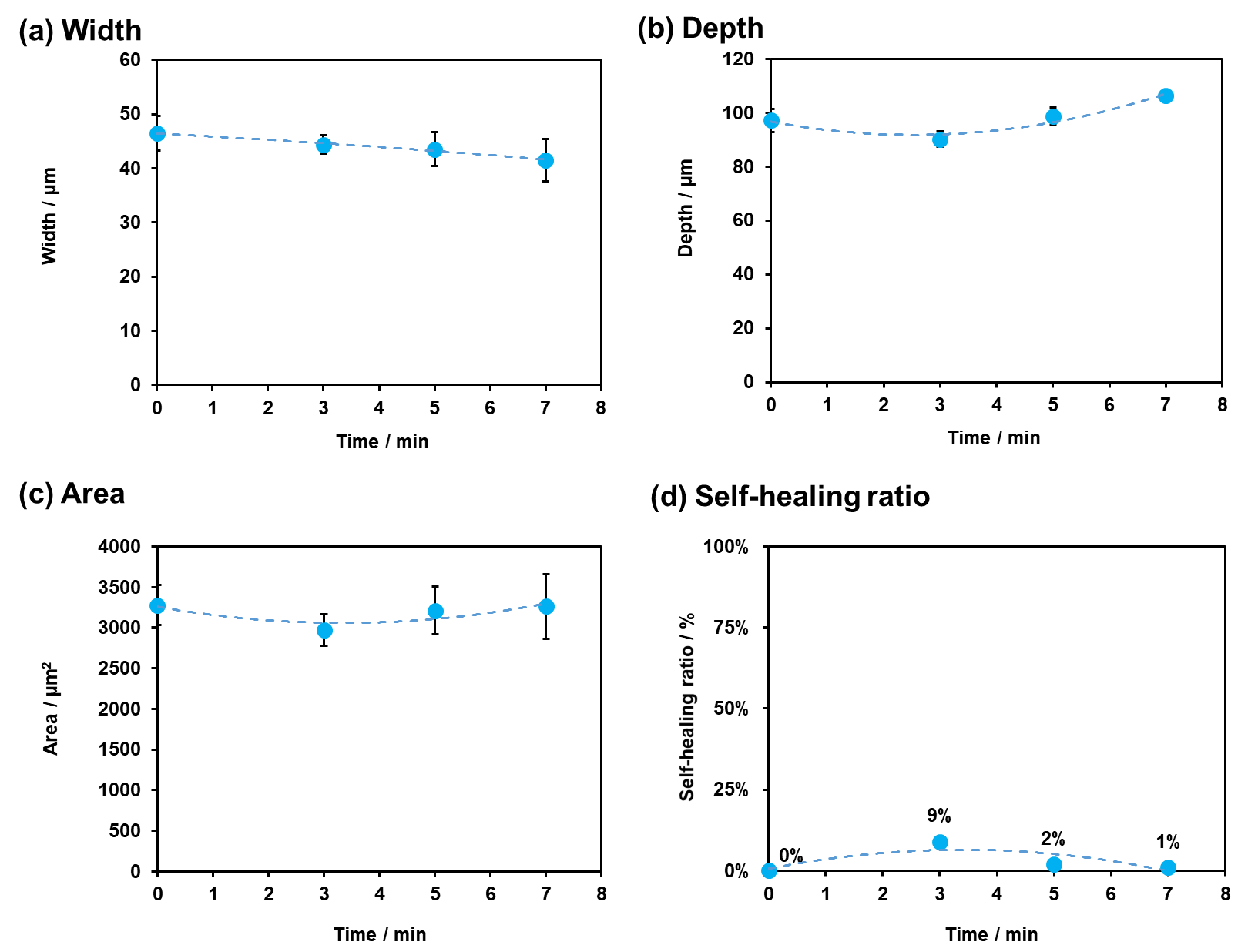


**Figure S17.** The scar profiles of HG(2.5) as (**a**) width, (**b**) depth, and (**c**) area. (**d**) The self-healing ratio of HG(2.5) calculated from the area. The error bar indicates sample standard deviation (n = 5).

**References**

1. Hocquelet, C.; Blu, J.; Jankowski, C.K.; Arseneau, S.; Buisson, D.; Mauclaire, L. Synthesis of calixarene–cyclodextrin coupling products. *Tetrahedron* **2006**, *62*, 11963-11971, doi:10.1016/j.tet.2006.09.089.
